# Modulation of Intestinal Epithelial Permeability in Differentiated Caco-2 Cells Exposed to Aflatoxin M1 and Ochratoxin A Individually or Collectively

**DOI:** 10.3390/toxins10010013

**Published:** 2017-12-27

**Authors:** Yanan Gao, Songli Li, Jiaqi Wang, Chaochao Luo, Shengguo Zhao, Nan Zheng

**Affiliations:** 1 Ministry of Agriculture Laboratory of Quality & Safety Control for Risk Assessment for Dairy Products (Beijing), Institute of Animal Science, Chinese Academy of Agricultural Sciences, Beijing 100193, China; gyn758521@126.com (Y.G.); Lisongli@caas.cn (S.L.); wang-jia-qi@263.net (J.W.); luochaochao839505@163.com (C.L.); zhaoshengguo1984@163.com (S.Z.); 2 Ministry of Agriculture-Milk and Dairy Product Inspection Center, Beijing 100193, China; 3 State Key Laboratory of Animal Nutrition, Institute of Animal Science, Chinese Academy of Agricultural Sciences, Beijing 100193, China

**Keywords:** aflatoxin M1, ochratoxin A, intestinal epithelial cells, tight junction, permeability

## Abstract

Aflatoxin M1 (AFM1) and ochratoxin A (OTA) are mycotoxins commonly found in milk; however, their effects on intestinal epithelial cells have not been reported. In the present study, we show that AFM1 (0.12 and 12 μM) and OTA (0.2 and 20 μM) individually or collectively increased the paracellular flux of lucifer yellow and fluorescein isothiocyanate (FITC)-dextrans (4 and 40 kDa) and decreased transepithelial electrical resistance values in differentiated Caco-2 cells after 48 h of exposure, indicating increased epithelial permeability. Immunoblotting and immunofluorescent analysis revealed that AFM1, OTA, and their combination decreased the expression levels of tight junction (TJ) proteins and disrupted their structures, namely, claudin-3, claudin-4, occludin, and zonula occludens-1 (ZO-1), and p44/42 mitogen-activated protein kinase (MAPK) partially involved in the mycotoxins-induced disruption of intestinal barrier. The effects of a combination of AFM1 and OTA on intestinal barrier function were more significant (*p* < 0.05) than those of AFM1 and OTA alone, yielding additive or synergistic effects. The additive or synergistic effects of AFM1 and OTA on intestinal barrier function might affect human health, especially in children, and toxin risks should be considered.

## 1. Introduction

Human exposure to mycotoxins not only can occur through direct dermal contact and inhalation of contaminated agricultural products, but also could occur through the consumption of foods of animal origin, such as milk and eggs, which were obtained from animals fed with mycotoxin-contaminated material [1]. Given that milk supplies the general public with a large quantity of essential nutrients, it is a part of the prevailing diet for all age groups. The largest consumers of milk are children because milk is vital for their growth and development. Thus, mycotoxin-contaminated milk can produce detrimental effects on their health [2]. Moreover, the presence of multiple mycotoxins in milk is likely. In raw milk from China, we identified several mycotoxins, including aflatoxin M1 (AFM1), ochratoxin A (OTA), zearalenone (ZEA), and α-zearalenol (α-ZOL) [3]. In raw bulk milk produced in northwest France in 2003, AFM1 was found in 3 out of 264 samples at levels of 26 ng/L or less, and OTA was detected in three samples at levels of 5 to 8 ng/L [4]. For infant milk formulas produced in Italy, AFM1 was found in 2 out of 185 samples (concentration range, 11.8 ng/L to 15.3 ng/L), while OTA was detected in 133 (72%) samples (concentration range, 35.1 to 689.5 ng/L) [5]. In a recent analysis of baby food (flours and milk powder) in Portugal, AFM1 and OTA were detected in 2 out of 27 samples, AFM1 was detected in two samples, OTA was detected in seven samples, and aflatoxin B1 (AFB1) and OTA were detected in one sample. For these samples, the AFM1 concentration was 0.017–0.041 μg/kg, and the OTA concentration was 0.034–0.212 μg/kg [6]. Considering the well demonstrated cytotoxic, genotoxic, and carcinogenic effects of AFM1, the International Agency for Research of Cancer (IARC) changed its carcinogenicity classification from Group 2 to Group 1 [7]. Furthermore, AFM1 is the only mycotoxin with an established maximum residue limit (MRL) in milk worldwide. The established MRL of AFM1 is 0.05 μg/kg in the European Union (EU) and 0.5 μg/kg in China and the United States of America (USA). We previously showed that OTA exerts a toxicity similar to that of AFM1 on human intestinal cells [8]. Furthermore, the IARC has classified OTA as a Group 2B carcinogen, suggesting that it is a possible human carcinogen [7]. Although there is no MRL for OTA in milk, a provisional tolerable weekly intake (PTWI) of 100 ng kg^−1^ body weight was established [9]. Hence, it is important to determine the toxicological effects of AFM1 and OTA on human health.

The gastrointestinal tract (GIT) is the first tissue barrier to come into contact with food contaminants, such as mycotoxins [10,11], and intestinal epithelial cells are most affected [12,13]. The GIT barrier is constituted by intercellular tight junction (TJ) proteins that localize to the apical domain of epithelial cells, where they selectively limit the passage of large molecules, ions, solutes, and water [14,15]. TJs are comprised of several multiprotein complexes that include transmembrane proteins (e.g., claudin, occludin, and junctional adhesion molecule (JAM) and cytoplasmic scaffolding proteins (e.g., zonula occludens (ZO)-1, ZO-2, and ZO-3) [16,17,18,19]. Therefore, it is likely that the defects in intestinal epithelial barrier function caused by mycotoxins are associated with the disruption of TJ integrity. Among the cell lines used as models of TJ function, the Food and Drug Administration (FDA) has recognized the Caco-2 cell line, originally isolated from human colon adenocarcinoma, as a reference model to assess the effects of drugs and toxins on intestinal barrier function [20]. A previous study showed that results obtained from this cell line are reproducible and applicable to in vivo data [21]. After culturing Caco-2 cells for 16–22 days, differentiated monolayers are established, mimicking the small intestine epithelial layer in that they form a polarized monolayer with functional TJs [22].

Presently, there is a growing awareness of the adverse effects of various mycotoxins on the intestine and the disruption of intestinal integrity in differentiated Caco-2 cells [23]. Romero et al. [24] reported that OTA decreased transepithelial electrical resistance (TEER) values and claudin-3, claudin-4, and occludin mRNA expression levels, and other studies demonstrated that treatment with OTA reduced intestinal barrier function in humans [25,26,27]. In addition, low concentrations of AFM1, added to either the apical or basal compartment of transwell chambers, significantly decreased TEER values [28]. The effects of mycotoxins on cells can be characterized as synergistic, additive, or antagonistic, and these effects might adversely affect human health. In addition, the contamination of aflatoxin B1 (AFB1) and OTA has been frequently observed in cereals and beans, and their concentrations in such foods are generally higher than that in milk. Wangikar et al. [29] demonstrated that AFB1 and OTA have antagonistic interaction in New Zealand White rabbits regarding their teratogenic effect. Huang et al. [30] reported that the mixture of AFB1, OTA, and ZEA exerted the greatest adverse effects on dairy goats, indicating the serious negative effects of the combinations of mycotoxins. We previously demonstrated synergistic and additive cytotoxic effects for OTA, ZEA, and/or α-ZOL with AFM1, except that AFM1 and ZEA produced an antagonistic effect on the proliferation of Caco-2 cells by isobologram analysis [8]. In recent decades, isobologram analysis has become the most commonly used approach to evaluate the interactive effects between chemical substances. Practical limitations exist in the combination analysis based on *Loewe Additivity* [31]. Estimation of dose–effect curves for the drugs being combined requires a certain amount of data and can rapidly become expensive as well as experimentally and computationally demanding, and makes the analysis of drug combination prohibitive [32]. *Loewe Additivity* model becomes unusable when a dose–effect curve is not available or difficult to model [33].

Previous studies have reported an association of epithelial barrier function with mitogen-activated protein kinases (MAPK) [34,35], which are responsible for intracellular signaling pathway. More importantly, MAPK are considered to play a vital role in inflammatory responses of epithelial cells and includes there subfamilies-p44/42 extracellular signal regulated kinase (ERK), p38 and c-Jun *N*-terminal Kinase (JNK). A mechanistic study demonstrated that the undermined epithelial barrier induced by deoxynivalenol (DON), which was reflected by the drop TEER values and reduced expression level of claudin-4 protein, was associated with the activation of ERK signaling pathway [36]. Though numerous studies have been reported that DON-induced intestinal barrier dysfunction was seen with the activation of MAPK signaling pathway, the underlying mechanisms of compromised intestinal barrier caused by AFM1 and OTA yet to be illustrated. With regard to intestinal barrier integrity, one study focused on the mycotoxin special DON, while another study examined the effects of DON and FB1 on the intestine of piglets [37,38]. Hence, it is crucial to understand the individual and combined effects of AFM1 and OTA on the structure and function of the gut. The purpose of this study was to investigate the individual and combined effects of AFM1 and OTA on intestinal permeability and to define the underlying mechanism(s). We hypothesized that (i) a combination of AFM1 and OTA, which frequently occur simultaneously in milk, might significantly affect intestinal permeability and TJ function and produce a variety of interactive effects, (ii) the decreased expression of TJ proteins leads to an increased epithelial permeability, and (iii) the decreased TJ proteins expression level mediated through modulation of MAPK. To address these hypotheses, we carried out TEER measurements, paracellular tracer flux assays, and related protein expression experiments in differentiated Caco-2 cells exposed to different concentrations of AFM1 and OTA individually and collectively.

## 2. Results

### 2.1. Effects of AFM1 and OTA Individually or Collectively on the TEER Values of Caco-2 Cell Monolayers

**Figure 1:**
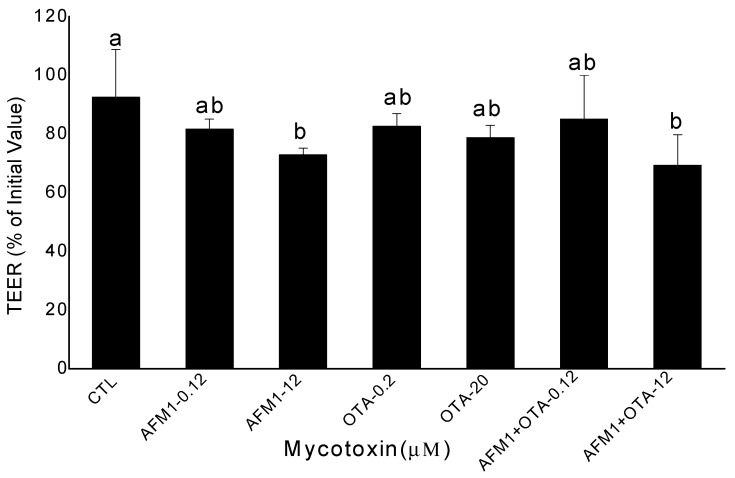
Changes in transepithelial electrical resistance (TEER) values in differentiated Caco-2 cells after treatment with different concentrations of aflatoxin M1 (AFM1) and ochratoxin A (OTA) individually or collectively (AFM1+OTA) for 48 h compared with the initial value. Results are expressed as the mean ± SEM of three independent experiments. TEER was measured before and after mycotoxin treatment for each condition. Different letters (a,b) indicate significant differences in TEER (*p* < 0.05). TEER, transepithelial electrical resistance. AFM1-0.12 represents AFM1 at 0.12 μM, AFM1-12 represents AFM1 at 12 μM, OTA-0.2 represents OTA at 0.2 μM, OTA-20 represents OTA at 20 μM, AFM1+OTA-0.12 represents the combination of AFM1 (0.12 μM) and OTA (0.2 μM), AFM1+OTA-12 represents the combination of AFM1 (12 μM) and OTA (20 μM).

The baseline TEER values of Caco-2 cell monolayers (on day 21 post-seeding) varied from 1411 to 1645 Ω·cm^2^. The culture of these monolayers in Dulbecco’s modified Eagle Medium (DMEM) containing 1% (v/v) methanol (control) did not significantly alter the initial TEER values over a period of 48 h. After 48 h of exposure to mycotoxins, the TEER values of Caco-2 cells exposed to non-cytotoxic concentrations of AFM1 (0.12 μM) and OTA (0.2 μM) individually or collectively were not significantly different (*p* > 0.05) from those of control cells. However, the TEER values of cells exposed to cytotoxic concentrations of AFM1 (12 μM) or the combination of AFM1 (12 μM) and OTA (20 μM) collectively were significantly lower (*p* < 0.05) than those of control cells. Furthermore, the TEER values of cells exposed to cytotoxic concentrations of AFM1 and OTA collectively were lower than those of cells exposed to AFM1 and OTA individually, although the difference between them were not significant (*p* > 0.05) (Figure 1).

### 2.2. Effects of AFM1 and OTA Individually or Collectively on the Permeability of Caco-2 Cell Monolayers

**Figure 2:**
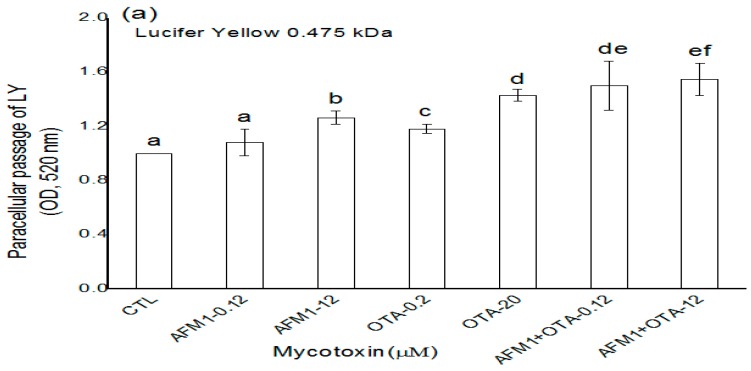

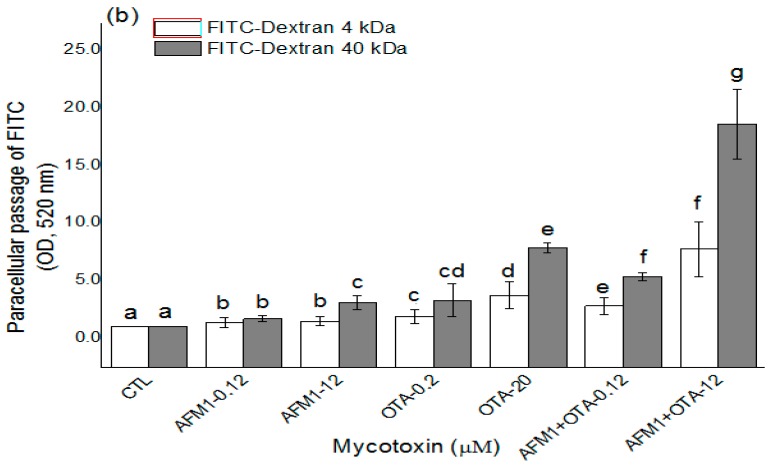
Mycotoxin increased the permeability of the Caco-2 cell monolayer. Caco-2 cells were cultured on transwell chambers and stimulated for 48 h with different concentrations of AFM1, OTA, and AFM1+OTA added to apical and basal compartments. Subsequently, the paracellular flux of lucifer yellow (LY) (0.457 kDa; (**a**)) and fluorescein isothiocyanate (FITC)-dextran (4 and 40 kDa; (**b**)) from the apical to the basal compartment was assessed. Results are expressed as the mean ± SEM of three independent experiments with three replicates. Different letters (a–g) indicate significant differences in FITC (*p* < 0.05). AFM1-0.12 represents AFM1 at 0.12 μM, AFM1-12 represents AFM1 at 12 μM, OTA-0.2 represents OTA at 0.2 μM, OTA-20 represents OTA at 20 μM, AFM1+OTA-0.12 represents the combination of AFM1 (0.12 μM) and OTA (0.2 μM), AFM1+OTA-12 represents the combination of AFM1 (12 μM) and OTA (20 μM).

The effects of non-cytotoxic and cytotoxic concentrations of AFM1 and OTA individually or collectively on intestinal permeability were investigated by measuring the paracellular flux of fluorescent tracers with different molecular weights (lucifer yellow (LY) and FITC-dextran) across Caco-2 cell monolayers. Except for the non-cytotoxic concentration of AFM1, which did not significantly affect (*p* > 0.05) the paracellular flux of LY (Figure 2a), mycotoxins significantly increased (*p* < 0.05) the paracellular flux of LY and FITC-dextran (4 and 40 kDa) across cell monolayers (Figure 2). For individual mycotoxins, the paracellular flux of LY and FITC-dextran (4 and 40 kDa) across cell monolayers was significantly higher (*p* < 0.05) after treatment with non-cytotoxic and cytotoxic concentrations of OTA than after similar concentrations of AFM1 (Figure 2). With regard to the combined use of AFM1 and OTA, both mycotoxins exerted a more significant effect (*p* < 0.05) on intestinal permeability than the individual mycotoxins (Figure 2).

### 2.3. Effects of ATM1 and OTA Individually or Collectively on TJ Protein Levels in Caco-2 Cell Monolayers

**Figure 3:**
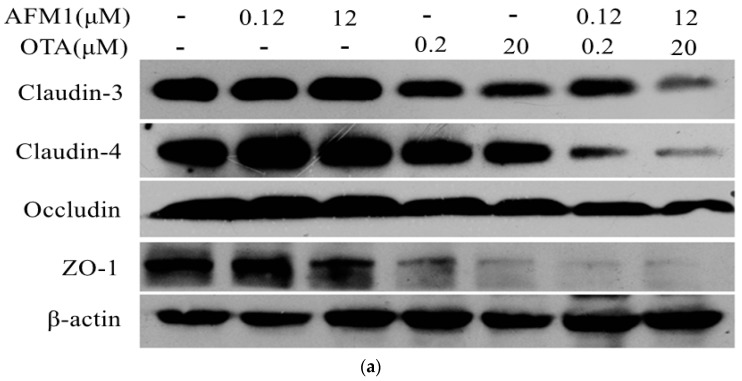

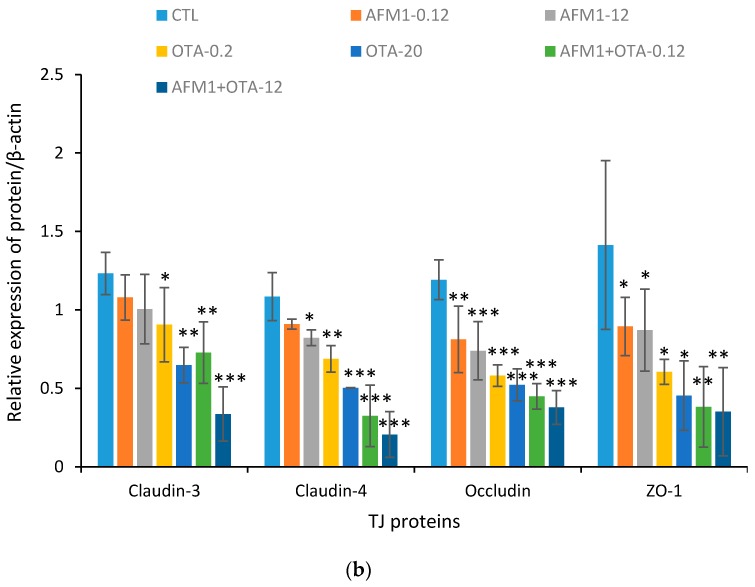
Effects of different concentrations of AFM1, OTA, and AFM1+OTA on TJ protein levels in Caco-2 cells after 48 h of exposure. (**a**) Cell proteins (20 μg) were separated by sodium dodecyl sulfate polyacrylamide gel electrophoresis (SDS-PAGE) and immunoblotted first for tight junction (TJ) proteins, and second for human β-actin; (**b**) the intensities of the TJ proteins were quantified with ImageJ software (Version 2.1.0, National Institutes of Health, Bethesda, MD, USA, 2006). Values were normalized to the loading control (human β-actin) and expressed relative to the negative control (untreated cells). Results are expressed as the mean ± Standard Error of Mean (SEM), *n* = 3–5. M-0.12 represents AFM1 at 0.12 μM, M-12 represents AFM1 at 12 μM, O-0.2 represents OTA at 0.2 μM, O-20 represents OTA at 20 μM, M+O-0.12 represents the combination of AFM1 (0.12 μM) and OTA (0.2 μM), M+O-12 represents the combination of AFM1 (12 μM) and OTA (20 μM). * *p* < 0.05; ** *p* < 0.001; *** *p* < 0.000.

To investigate the mechanism by which mycotoxins increase the permeability of Caco-2 cell monolayers, the levels of TJ proteins were quantified by Western blot analysis. Occludin and ZO-1 levels in Caco-2 cells exposed to non-cytotoxic and cytotoxic concentrations of AFM1 and OTA individually or collectively were significantly lower (*p* < 0.05) than those in control cells. By contrast, claudin-3 and claudin-4 levels in cells exposed to OTA, but not ATM1, or both mycotoxins were significantly lower (*p* < 0.05) than those in control cells (Figure 3). The negative effects of OTA on the levels of claudin, occludin, and ZO-1 were significantly greater (*p* < 0.05) than those of AFM1. Furthermore, the negative effects of AFM1 and OTA collectively were greater than those of AFM1 and OTA individually. The decrease in claudins, occludin, and ZO-1 expression in cells exposed to non-cytotoxic concentrations of AFM1 and OTA was not significantly different (*p* > 0.05) from that in cells exposed to cytotoxic concentrations.

### 2.4. Effects of AFM1 and OTA Individually or Collectively on TJ Protein Localization in Caco-2 Cell Monolayers

**Figure 4:**
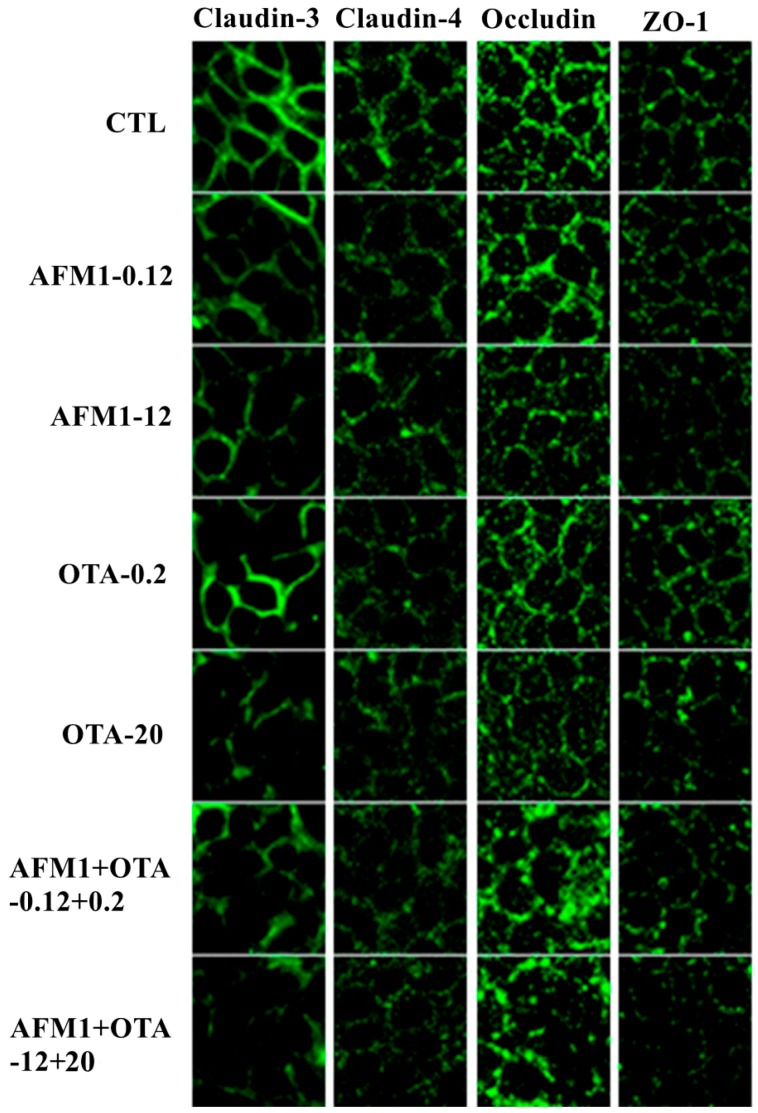
Effects of AFM1 and OTA on the localization of TJ proteins. Differentiated Caco-2 cells were cultured on transwell chambers for 21 days. The cells were then treated for 48 h with different concentrations of AFM1 and OTA added to apical and basal compartments, followed by fixation and staining with an anti-claudin-3, anti-claudin-4, anti-occludin, or anti-zonula occludens-1 (ZO-1) antibody, as described in Section 2.

The localization of TJ proteins was assessed by immunofluorescent staining. In confluent Caco-2 cells not exposed to mycotoxins, claudin-3, claudin-4, occludin, and ZO-1 localized at the plasma membrane and resembled a cobblestone pattern. There was no significant difference in the localization of claudin-3 and ZO-1 in cells exposed to non-cytotoxic concentrations of mycotoxins individually or collectively. Furthermore, claudin-3 and ZO-1 were hardly detected in cells exposed to cytotoxic concentrations of AFM1 and OTA individually or collectively, suggesting that claudin-3 and ZO-1 protein synthesis was affected. Mycotoxins also affected claudin-4, as evidenced by a faint immunofluorescent signal and a discontinuous cobblestone pattern unlike in control cells. Although there was no difference in the localization of occludin in cells exposed to a non- or cytotoxic concentration of AFM1 and a non-cytotoxic concentration of OTA, a cytotoxic concentration of OTA and non- or cytotoxic concentrations of AFM1 and OTA disrupted the continuous cobblestone pattern. Indeed, the individual use of mycotoxins did not significantly affect the localization of occludin in differentiated Caco-2 cells, while the combined use of mycotoxins, especially at cytotoxic concentrations, undermined the integrity of the cobblestone pattern of occludin (Figure 4).

### 2.5. The Interactive Effects of the Combination of AFM1 and OTA

**Figure 5:**
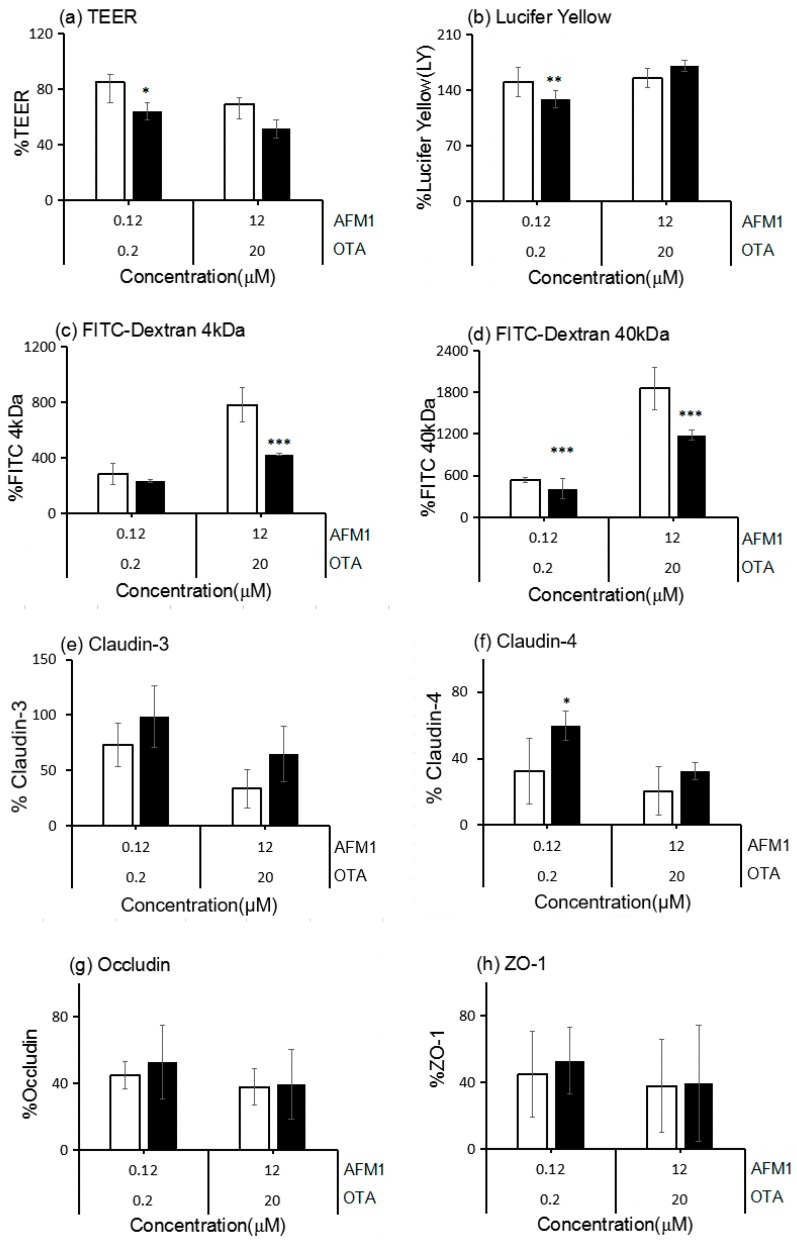
Interactive cytotoxic effects of binary combinations of AFM1 and OTA in differentiated Caco-2 cells after exposure for 48 h (**a**–**h**). After the exposure to mycotoxins, TEER, FITC-dextran (4 and 40 kDa) paracellular flux, and TJ protein expression were measured. There were no significant interactive effects for claudin-3, occludin, and ZO-1 expression. Data are expressed as a percentage of the untreated control for each parameter. White bars represent the measured values, and dark bars represent the expected values. * *p* < 0.05; ** *p* < 0.001; *** *p* < 0.000 represent both significant synergistic and antagonist effects.

After assessing the interactive effects of AFM1 and OTA at different concentrations on TEER, according to the analysis for interactions described in Section 4.10, we found an antagonistic effect between non-cytotoxic concentrations of AFM1 and OTA, and the measured value was 20.8% (*p* < 0.05) higher than the expected value. However, an additive effect was found at cytotoxic concentrations of the mycotoxins, with a non-significant difference (*p* > 0.05) between the measured and expected values (Figure 5a).

Synergistic effects were observed for the paracellular flux of LY after treatment with non-cytotoxic concentrations of AFM1 and OTA collectively (with a 21.5% difference over the expected values, *p* < 0.05), the paracellular flux of FITC-dextran (4 kDa) after treatment with cytotoxic concentrations of both mycotoxins (with a 357.0% difference over the expected values, *p* < 0.05), and the paracellular flux of FITC-dextran (40 kDa) after treatment with non-cytotoxic and cytotoxic concentrations of both mycotoxins (with a 129.8% and 679.0% difference over the expected values, respectively, *p* < 0.05) (Figure 5b–d, respectively). Additive effects were observed for the paracellular flux of LY after treatment with cytotoxic concentrations of both mycotoxins (*p* > 0.05) and the paracellular flux of FITC-dextran (4 kDa) after treatment with non-cytotoxic concentrations of both mycotoxins (*p* > 0.05) (Figure 5b,c, respectively).

The additive effects of AFM1 and OTA were evident on claudin-3, occludin, and ZO-1 expression, with no significant difference between the measured and expected values (Figure 5e,g,h). Synergistic effects were observed for claudin-4 expression after treatment of Caco-2 cells with a non-cytotoxic concentration of both mycotoxins, with a 27.4% (*p* < 0.05) decrease compared with the expected values, and additive effects were shown at cytotoxic concentrations of both mycotoxins (Figure 5f).

### 2.6. Correlations between TJ Junction and Intestinal Permeability

**Figure 6:**
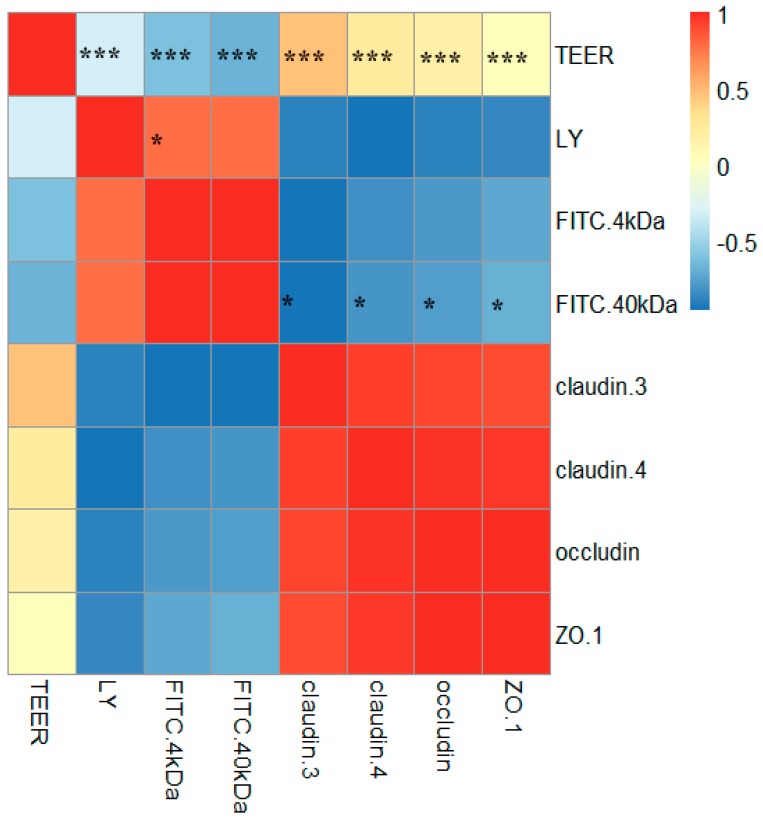
Heat map showing correlations among TEER, FITC-dextran (4 and 40 kDa), and TJ proteins (claudin-3, claudin-4, occludin, and ZO-1) for differentiated Caco-2 cells taking into account data from all of the experiments performed in this study. The heat map is a visual representation of correlated values between each pair of parameters denoted by the corresponding row and the column of the matrix. Red represents a positive correlation, yellow represents a low correlation, and blue represents a negative correlation, as shown in the color key. Statistical significance was analyzed by Spearman’s correlations. The number scale to the right represents the correlation coefficients. The higher the number, the higher the correlation. * *p* < 0.05 and *** *p* < 0.000. (For an interpretation of the references to the color in this figure, the reader is referred to the web version of the article.).

To understand the significance of the changes in TJ protein levels and intestinal permeability following exposure to mycotoxins, the correlations among TEER values, the permeability of fluorescent tracers (LY, FITC-dextran (4 kDa), and FITC-dextran (40 kDa)), and the levels of TJ proteins (claudin-3, claudin-4, occludin, and ZO-1) were evaluated. Significantly negative (*p* < 0.000) correlations were found between the TEER values and the three fluorescent tracers, and a significantly positive (*p* < 0.05) correlation was found between LY and FITC-dextran (40 kDa). In addition, there were significantly positive (*p* < 0.000) correlations between the TEER values and the four TJ proteins. There were also significantly negative (*p* < 0.05) correlations between FITC-dextran (40 kDa) and the four TJ proteins, although there were no significant (*p* > 0.05) correlations between LY or FITC-dextran (4 kDa) and claudin-3, claudin-4, occludin, or ZO-1. The significant correlations between the TEER values or FITC-dextran (40 kDa) and the TJ proteins suggest that an increased epithelial permeability associates with the disruption of TJ integrity (Figure 6).

To further investigate the role of TJs in intestinal epithelial permeability, we silenced occludin, a representative TJ protein, in Caco-2 cells. Occludin knockdown in transfected cells was confirmed by immunoblotting (Figure S1a). More specially, the expression level of occludin of siRNA group were significantly lower than that in control and NC group, indicating that the effects of occludin knockdown was creditable. In addition, the TEER values in occludin-silenced cells were significantly lower (*p* < 0.05) than those in control and NC-silenced cells (Figure S1b), indicating that occludin plays an important role in TJ integrity. These results also indicate that TJs contribute to the maintenance of epithelial permeability.

### 2.7. AFM1 and OTA Induce Barrier Dysfunction via MAPK-Dependent Mechanism

**Figure 7:**
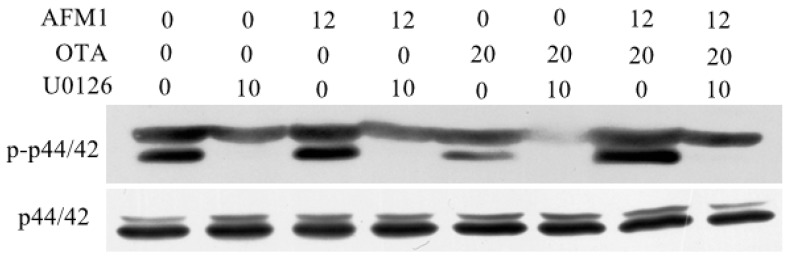
U-0126 monoethanolate (U-0126) (10 μM) counteracted AFM1 (12 μM) and OTA (20 μM)-induced p44/42 mitogen-activated protein kinase (MAPK) (extracellular regulated protein kinases 1/2, [ERK] 1/2). Differentiated Caco-2 cells were either pretreated with U-0126 or complete cultivation medium for 1 h before addition of individual and combined AFM1 and OTA for 30 min. Phosphorylation of p44/42 MAPK was determined by immunoblotting.

In order to identity intracellular signaling pathways related to AFM1 and OTA-induced downregulation of TJ proteins, the MAPK pathway was measured in differentiated Caco-2 cells treated with these mycotoxins individually or collectively. Cells were pretreated specific pharmacological inhibitors for the related molecules (p44/42 (U-0126), JNK (SP600125), p38 (SB203580)) for 1 h, followed by a 30 min exposure of AFM1 and OTA. The immunoblotting results showed that the combined mycotoxins led to a more significant activation of p44/42 MAPK than these individually and the pretreatment of inhibitor U-0126 prevented individual and combined AFM1 and OTA-induced p44/42 MAPK phosphorylation (Figure 7), while the mycotoxin-induced phosphorylation of p38 and JNK MAPK was not affected by the corresponding inhibitors (data not shown).

## 3. Discussion

It is common to find co-contamination of mycotoxins in milk, which has been reported worldwide, instead of the occurrence of individual mycotoxins [3]. However, most studies mainly focused on the toxic effects of individual mycotoxins. Given that the intestine functions as the initial barrier against mycotoxins, and that it could be exposed to high doses of these toxins, little is known about the interaction of mycotoxins with the human intestinal epithelium [39]. Thus, it is essential to determine the combined effects of mycotoxins on the intestinal barrier. In this study, we investigated the effects of AFM1 and OTA, which are common mycotoxins found in milk, on differentiated Caco-2 cells that resemble epithelial cells of the small intestine to determine the underlying mechanisms of the dysfunction of the intestinal barrier caused by mycotoxins and to discover whether they have additive, synergistic or antagonistic effects.

TEER values and the paracellular flux of tracers (LY, FITC-dextran) are vital parameters in the study of epithelial barrier integrity. In this study, a dose-dependent decrease in TEER of the differentiated Caco-2 monolayer was observed after 48 h of AFM1 and/or OTA treatment. This is in agreement with previous studies [28,40,41,42] that reported individual mycotoxins, such as AFM1, AFB1, OTA, patulin (PAT), and FB1, to decrease TEER values in Caco-2 cells and to disrupt barrier function. A previous study has illustrated that a reduction in TEER can however be caused by different events including: (i) increase in paracellular permeability to ions; (ii) changes in transcellular ion flux through altered plasma membrane channels or pumps; or (iii) uncontrolled cell death within the monolayer [43]. From this angle, the reduced cell viability induced by AFM1 and OTA at higher concentration may play a role in the change of TEER values and cell monolayer permeability. Indeed, after 48 h of exposure, AFM1 and OTA individually or collectively induced a significant increase in the translocation of LY and FITC-dextran from the apical to the basal compartment. These findings are consistent with the data of Akbari et al. [44] and Pinton et al. [45], demonstrating that DON increased the paracellular permeability to FITC-dextran in Caco-2 cells and intestinal epithelial cell lines from porcine (IPEC-1) cells.

TJs are comprised of multiple protein complexes located at the apical domain of lateral membranes of intestinal epithelial cells. The integral components of TJ complexes are the four transmembrane proteins, namely, occludin, claudins, JAMs, and tricellulin, as well as ZO-1, which anchors the transmembrane proteins to the perijunctional actomyosin ring [15]. To our best knowledge, there are few studies that report the effects of AFM1 or OTA on the integrity of the intestinal barrier. Romero et al. [24] showed that, after 24 h of exposure to OTA, claudin-3, claudin-4, and occludin mRNA expression decreased in differentiated Caco-2 cells. McLaughlin et al. [42] reported that OTA reduced claudin-3 and claudin-4 protein levels after 24 h of exposure. Although there was a moderate decrease in TEER values in the presence of low concentrations of AFM1 added to either the apical or basal compartment of transwell chambers, occludin and ZO-1 localization was relatively unchanged in Caco-2 cells [28]. By Western blotting and immunofluorescent microscopy, we demonstrate for the first time the loss of TJ proteins (claudin-3, claudin-4, occludin, and ZO-1) from cells. Furthermore, the emphasis for Western blotting and immunofluorescent staining of tight junction proteins are different. The focus of Western blotting analysis is to measure the change of proteins expression level induced by mycotoxins, while the aim of immunofluorescent staining is to determine the effect of mycotoxins on the cellular localization of cell adhesion molecules. Thereby, the different objective of these two experiments could explain that the trend between Figure 3 and Figure 4 is not closely coincident. The marked loss of ZO-1 could be explained by the fact that ZO-1 links TJ proteins to the actin cytoskeleton, which is critical for the maintenance of the structure and function of the TJ barrier. Defective barrier integrity may contribute to various intestinal and systemic inflammatory diseases. Clinical studies showed that, in patients with Crohn’s disease (CD), the expression of claudin-3 and occludin was decreased [46]. Thus, further studies are needed to understand the mechanisms behind this AFM1/OTA-induced decrease in TJ permeability.

There is considerable evidence showing that disrupted intestinal permeability is related to the decreased expression and translocation of TJ proteins. Watari et al. [47] demonstrated that homoharringtonine (HHT) increased the paracellular flux of FITC-dextrans (4 and 40 kDa) and reduced the protein levels of claudin-3, claudin-5, and claudin-7 in Caco-2 cells. Akbari et al. [41] reported that DON increased the paracellular flux of LY and FITC-dextran (4 kDa) and decreased the levels of claudin-1, claudin-3, and claudin-4. Romero et al. [24] reported decreased TEER values with decreased claudin-3 and occludin mRNA expression levels in Caco-2 cells exposed to AFB1, FB1, T2, and OTA at concentrations up to 100 μM for seven days. In the present study, the increased intestinal permeability caused by AFM1 and OTA was concomitant with the reduced expression of claudin-3, claudin-4, occludin, and ZO-1. Indeed, significant correlations were observed between TEER values/FITC-dextran (40 kDa) and the four TJ proteins, which were assessed by Spearman’s correlations. Therefore, we concluded that the mechanism by which these mycotoxins reduce the barrier properties of Caco-2 cells appears to be via altering the expression levels and/or distribution of specific TJ proteins, namely, claudin-3, claudin-4, occludin, and ZO-1.

Emerging evidence have demonstrated involvement of MAPK signaling in the compromised barrier function caused by TJ proteins. For example, the reduced expression of occludin and ZO-1 in human gastric epithelial cells may underlie clopidogrel-induced gastric mucosal injury, which involves the activation of the p38 MAPK pathway [48]. DON-induced MAPK activation decreased the expression of claudin in the disrupted intestinal barrier [36]. Recently, one study reported that arsenic downregulated TJ protein claudin through p38 MAPK in HT29 intestinal epithelial cell line [49]. Thus, it is reasonable to conclude that mycotoxin-induced MAPK pathway activation decreases the expression of TJ proteins, which in turn reduces the barrier function of the intestine as evaluated by TEER and paracellular permeability. In the present study, we examined the extent to which the activation of MAPK regulate barrier disruption of AFM1 and OTA. The results showed that only p44/42 MAPK play an important role in mediating the expression level of TJs, especially for their combination, and this finding is in accordance with literature reporting the regulation of TJs structure and function is often regulated by this MAPK [50]. To further investigate whether p44/42 activation mediates intestinal barrier effects of AFM1 and OTA individually or collectively, differentiated Caco-2 cells exposed to these mycotoxins in the absence and presence of U-0126. According to our study, pretreatment with U-0126 in differentiated Caco-2 cells and then subsequent with mycotoxins in the presence of the inhibitor significantly reduced the adverse effects induced by AFM1 and OTA individually or collectively, indicating that the activation of p44/42 MAPK is partially involved in these mycotoxins-induced barrier disruption.

One of the aims of the present study was to assess the combined effects of AFM1 and OTA. In this study, non-cytotoxic concentrations of AFM1 and OTA exerted antagonistic effects on the TEER, while these mycotoxins at the same concentrations exerted synergistic effects on the paracellular flux of LY and FITC-dextran (40 kDa) and the expression of claudin-4. All other interactions of AFM1 and OTA at different concentrations with regard to epithelial permeability were additive. It is commonly assumed that mycotoxins with the same mode of action and/or the same cellular target exert a synergistic or additive effect when present together [51]. Studies demonstrated that exposure to AFM1 and OTA resulted in oxidative DNA damage, which represents the predominant mechanism for cytotoxicity [52,53,54]. This might explain the synergistic and additive interaction effects that we observed in this study. The antagonistic effect of AFM1 and OTA may be explained by the competition for glutathione (GSH) in cells. Since electrophiles generated from the metabolism of OTA with hydroquinone–quinone reduce GSH to produce GSH conjugates, the hydroxyl group of AFM1 can also be conjugated with GSH [55,56,57]. Indeed, the interactive effects were different depending on the concentrations and exposure time used, the endpoint assessed, or the species applied. Furthermore, the deeper mechanisms underlying the mycotoxin-induced dysfunction of intestinal barrier need to be explored.

In summary, we herewith present a detailed in vitro investigation of AFM1 and OTA individually or collectively on intestinal barrier integrity. Furthermore, the present study for the first time shows that AFM1 and OTA impaired the intestinal barrier as evaluated by increased TEER values and permeability tracer flux, which was associated with the perturbation of TJ complexes, including claudins, occludin and ZO-1, and p44/42 MAPK at least partially involved in the detrimental effects of integrity of TJs caused by AFM1 and OTA. Indeed, AFM1 and OTA exerted different interactive effects, depending on the endpoints of intestinal permeability assessed. These findings may explain some of the observed adverse effects on the GIT in vivo caused by these mycotoxins. The special molecular mechanisms that p44/42 MAPK influence the mycotoxin-mediated TJ disruption need to be clarified and further studies would be required to confirm these finding in vivo cases.

## 4. Materials and Methods

### 4.1. Chemicals and Reagents

AFM1 (structural formula, C_17_H_12_O_7_; molecular weight, 328) and OTA (structural formula, C_20_H_18_ClNO_6_; molecular weight, 403) were purchased from Fermentek, Ltd. (Jerusalem, Israel). Mycotoxins were dissolved in methanol as previously described [58,59]. AFM1 and OTA were dissolved in methanol to final concentrations of 400 μg/mL and 5000 μg/mL, respectively. Both stock solutions were stored at −20 °C. Dulbecco’s modified Eagle medium (DMEM), fetal bovine serum (FBS), antibiotics (100 units/mL penicillin and 100 μg/mL streptomycin), nonessential amino acids (NEAA) were purchased from Life Technologies (Carlsbad, CA, USA). An Enhanced Cell Counting Kit-8, radio immunoprecipitation assay (RIPA) lysis buffer, immunofluorescent staining blocking buffer, primary and secondary antibody dilution buffer were purchased from Beyotime Biotechnology (Shanghai, China). Protease inhibitors, lucifer yellow (LY) and fluorescein isothiocyanate (FITC)-dextran were purchased from Sigma-Aldrich (St. Louis, MO, USA). Rabbit anti-claudin-3 and rabbit anti-claudin-4 were purchased from Abcam (Cambridge, MA, USA). Rabbit anti-ZO-1 and rabbit anti-occludin were purchased from Thermo Scientific (Waltham, MA, USA). Rabbit anti-β-actin was purchased from Cell Signaling Technology (Trask Lane Danvers MA, USA). Goat anti-rabbit IgG conjugated to horseradish peroxidase and Alexa Fluor 488 mouse anti-rabbit IgG were purchased from Bioss Antibodies (Beijing, China). Three MAPK-specific inhibitors (SB-203580, U-0126 and SP-600125) were purchased from Sigma-Aldrich, and their stock solutions were prepared with dimethyl sulfoxide (DMSO).

### 4.2. Cell Culture and Differentiation

The human colon adenocarcinoma Caco-2 cell line (passage number, 18) was obtained from American Type Culture Collection (ATCC) (Manassas, VA, USA). The cells were cultured in DMEM containing 4.5 g/L glucose, 10% FBS, antibiotics (100 units/mL penicillin and 100 μg/mL streptomycin), and 1% NEAA at 37 °C in a humidified atmosphere of 5% CO_2_ in air.

In the present study, Caco-2 cells plated at a passage number (passage number, 23–35) similar to that reported by Watari et al. [47] were cultured in 6- or 12-well transwell chambers (Corning, NY, USA) at a density of 4 × 10^4^ cells/cm^2^, and the medium was replaced every other day until 21 days. The mean TEER value was 1528 ± 117 Ω·cm^2^ (Figure S2), which exceeded the TEER cut-off value of 300 Ω·cm^2^, as measured by a Millicell-ERS volt-ohm meter (Millipore, Temecula, CA, USA). The differentiated Caco-2 cells were then used for TEER measurement, permeability tracer flux assay, Western blot analysis, and immunofluorescent staining.

### 4.3. Cytotoxicity Assay

The effects of mycotoxins on the proliferation of intestinal cells were determined using the Enhanced Cell Counting Kit (CCK)-8 according to the manufacturer’s instructions. Caco-2 cells were seeded at 6 × 10^4^ cells/well in 100 μL of complete proliferation medium in 96-well plates. After 24 h of culture, AFM1 (0.012, 0.12, 1.2, 6, and 12 μM) and OTA (0.02, 0.2, 2, 10, and 20 μM), which were prepared by adding DMEM, were added. After 48 h, 100 μL of CCK-8 Reagent was added per well, and the cells were incubated for 2 h. The absorbance was measured at 450 nm using an automated ELISA reader (Thermo Scientific, Waltham, MA, USA). Results were expressed as the percentage of cell survival (%) with respect to the control. Based on the cell viability results (Figure S3), 0.12 μM was selected as the non-cytotoxic AFM1 concentration and 12 μM as the cytotoxic concentration, and the corresponding concentrations of OTA (0.2 and 20 μM) were chosen. In all subsequent experiments, AFM1 at 0.12 and 12 μM, and OTA at 0.2 and 20 μM, were used.

### 4.4. TEER Measurement

The measurement of TEER across epithelial cell monolayers is one of the best ways to evaluate the integrity of the TJ barrier in Caco-2 models [20]. Caco-2 cells were cultured on transwell chambers as described in Section 4.2. Cells were challenged for 48 h with increasing concentrations of AFM1 (0.12 and 12 μM) or OTA (0.2 and 20 μM), which were added to apical and basal compartments of transwell chambers. Binary combinations (AFM1/OTA) were also tested using identical concentrations and exposure time. Results were expressed relative to the initial TEER value for each insert and presented as the mean ± standard error of the mean (SEM) of five independent experiments.

### 4.5. Permeability Tracer Flux Assay

In addition to TEER measurement, the paracellular flux of tracers across the cell monolayer also reflects the permeability of intestinal barrier. The most common paracellular tracers used in in vitro models are fluorescent compounds (e.g., LY) or fluorescently labeled compounds (e.g., FITC-dextran and FITC-inulin). To determine whether mycotoxins individually and collectively affect the size selectivity of the permeability barrier, different molecular size tracers were used. Caco-2 cell monolayers were cultured on transwell chambers to confluency and treated with AFM1 and OTA individually or collectively for 48 h as described in Section 4.4. The membrane-impermeable tracers LY and FITC conjugated-dextran with a molecular mass of 4 or 40 kDa were dissolved in phosphate-buffered saline (PBS) to a final concentration of 100 μg/mL. In this study, these tracers were added to the apical compartment for 4 h. The fluorescent intensity in the basal compartment was measured with an automated ELISA reader. The excitation and emission wavelengths were 410 and 520 nm, respectively, for LY, and 490 and 520 nm for FITC-dextran.

### 4.6. Western Blot Analysis

Immunoblot assays were conducted to examine the expression of TJ proteins claudin-3, claudin-4, occludin and ZO-1 as well as the phosphorylation status of MAPK proteins ERK, p38 and JNK. For TJ proteins analysis, Caco-2 cell monolayers were cultured on transwell chambers and incubated for 48 h with increasing concentrations of AFM1 and OTA, which were added to apical and basal compartments. Caco-2 cells were lysed with RIPA lysis buffer containing protease inhibitors. Equal amounts of protein were combined with a nonreducing buffer, heat-denatured, electrophoresed on sodium dodecyl sulfate (SDS)-polyacrylamide gels, and electroblotted onto polyvinylidene difluoride membranes. The membranes were blocked with 5% skim milk in PBS for 1.5 h at room temperature. Subsequently, rabbit anti-claudin-3, rabbit anti-claudin-4, rabbit anti-ZO-1, rabbit anti-occludin, and rabbit anti-β-actin antibodies were diluted according to the manufacturers’ instructions and incubated with membranes for 3 h at room temperature. Goat anti-rabbit IgG conjugated to horseradish peroxidase was applied for 1 h at room temperature. Peroxidase activity was visualized on a radiographic film using enhanced chemiluminescence reagents. Signal intensities were determined by densitometry using ImageJ 2× software (Version 2.1.0, National Institutes of Health, Bethesda, MD, USA, 2006), and values were normalized to the loading control (human β-actin). In the kinase phosphorylation assay, after 21 days for culturing differentiated Caco-2 cells, the cells were treated with serum-free medium for 12 h. After 12 h, cells were washed and treated first with or without 10 μM three inhibitors for 1 h and then with 12 μM AFM1 or/and 20 μM OTA for 30 min. Whole cell lysates were collected, normalized for total proteins, and analyzed for the levels of phosphorylated ERK, p38 and JNK proteins.

### 4.7. Immunofluorescent Staining

The localization of TJ proteins was assessed by confocal microscopy. Confluent Caco-2 cells were incubated for 48 h with AFM1 and OTA individually or collectively by adding to apical and basal compartments. The cells were fixed with 4% paraformaldehyde for 10 min at 4 °C and then permeabilized with 0.1% Triton X-100 in PBS for 5 min. The cells were incubated in blocking buffer for 1.5 h, followed by incubation with an anti-claudin-3, anti-claudin-4, anti-occludin, or anti-ZO-1 antibody diluted in primary antibody dilution buffer for 1.5 h. The cells were then incubated with Alexa Fluor 488 mouse anti-rabbit IgG diluted in secondary antibody dilution buffer for 45 min at 37 °C in the dark. The cells were observed under a LSM780 immunofluorescent microscope (Carl Zeiss, Inc., Thornwood, NY, USA).

### 4.8. Downregulation of Occludin by siRNA

Occludin small interfering RNA (occludin siRNA; GenePharma) and negative control (NC) siRNA were transiently transfected into differentiated Caco-2 cells using OptiMEM siRNA transfection medium and Lipofectin 2000 siRNA transfection reagent according to the manufacturer’s instructions. The primer sequences were GCGUUGGUGAUCUUUGUUATT (sense) and UAACAAAGAUCACCAACGCTT (antisense). The transfection medium containing the siRNA and siRNA transfection reagent was mixed with serum-free culture medium and incubated with the differentiated Caco-2 cells. The medium was replaced after 6 h, and cells were incubated for an additional 24 h, and then TEER values were determined. Cells were then collected and lysed, and occludin expression was examined.

### 4.9. Comparison between Expected and Measured Endpoints

To compare the expected values (expressed as %) with the measured values (expressed as %), the expected value was calculated by the addition of the mean after exposure to one (or two) substance(s) with the mean value obtained after exposure to the second or third substance [60]:

mean (expected for AFM1+OTA) = mean (AFM1) + mean (OTA) − 100%,(1)

% difference = |mean (expected for AFM1+OTA) − mean (measured for AFM1+OTA)|,(2)

Taking the TEER value from the combined use of AFM1 and OTA at non-cytotoxic concentrations as an example, the mean measured TEER value was 84.9%, and, according to the calculation, the mean expected TEER value was 64.1%. Hence, the % difference was 20.8%:

SEM (expected for AFM1+OTA) = [(SEM for AFM1)^2^ + (SEM for OTA)^2^]^1/2^, (3)

### 4.10. Analysis for Interactions and Correlations

We previously used isobologram analysis, which is a quantitative method to measure the interaction of the combined use of mycotoxins [8]. However, the weakness of this approach is that it requires specialized software to calculate the combination index (CI) value. To analyze the interactive effects without this technical tool, expected and measured endpoints were compared.

The impaired intestinal barrier led to the situation that TEER values, as well as the expression of claudin-3, claudin-4, occludin, and ZO-1, decreased, while LY, FITC-4 kDa, and FITC-40 kDa increased. Synergistic effects were defined as those produced by various chemicals that were greater than the sum of their individual effects. Antagonistic effects were defined as those produced by various chemicals that were lower than the sum of each role.

The significance of difference in expected and measured values was calculated using an unpaired *t*-test, with *p* < 0.05 being considered statistically significant. The results were interpreted as follows:

Additive effects were defined as measured values for endpoints that were not significantly above or below the expected values (*p* > 0.05).

Synergistic effects were defined as measured values that were significantly lower than the expected values for endpoints TEER, claudin-3, claudin-4, occludin, and ZO-1 and significantly higher than for endpoints LY, FITC-4 kDa, and FITC-40 kDa.

Antagonistic effects were defined as measured values that were significantly higher than the expected values for endpoints TEER, claudin-3, claudin-4, occludin, and ZO-1 and significantly lower than the endpoints LY, FITC-4 kDa, and FITC-40 kDa.

Correlations among TEER values, the paracellular flux of LY, FITC-dextran (4 kDa), or FITC-dextran (40 kDa), and the levels of claudin-3, claudin-4, occludin, or ZO-1 in differentiated Caco-2 cells treated with AFM1 and OTA individually or collectively were assessed by Spearman’s correlations (nonparametric).

### 4.11. Statistical Analysis

Statistical analysis was performed using SAS 9.2 software (Cary, NC, USA). Data were expressed as the mean ± SEM of three independent experiments. Differences between groups were analyzed by one-way analysis of variance (ANOVA), followed by the Tukey Honestly Significant Difference (HSD) test for multiple comparisons. Statistically significant cytotoxic effects are represented by * *p* < 0.05; ** *p* < 0.001; and *** *p* < 0.0001.

## Supplementary Materials

The following are available online at www.mdpi.com/2072-6651/10/1/12/s1, Figure S1: Mycotoxins disrupt intestinal epithelial permeability by affecting occludin expression, Figure S2: Transepithelial electrical resistance values (Ω × cm^2^) in differentiated Caco-2 cells were measured at different time points until 21 days, Figure S3: Concentration–response bar charts for AFM1 and OTA in Caco-2 cells after 48 h of exposure. Results are expressed as the mean ± SEM of three independent experiments with five replicates.
