# Supplementary material for: Modulation of Intestinal Epithelial Permeability in Differentiated Caco-2 Cells Exposed to Aflatoxin M1 and Ochratoxin A Individually or Collectively
Source: Toxins (Basel). 2017 Dec 27;10(1):13. doi: 10.3390/toxins10010013 (PMC5793100; doi:10.3390/toxins10010013)
Supplement: Supplementary file 1 [file toxins-10-00013-s001.pdf]

Supplementary Materials: Modulation of Intestinal Epithelial Permeability in Differentiated Caco-2 Cells Exposed to Aflatoxin M1 and Ochratoxin A Individually or Collectively

Yanan Gao, Songli Li, Jiaqi Wang, Chaochao Luo, Shengguo Zhao and Nan Zheng

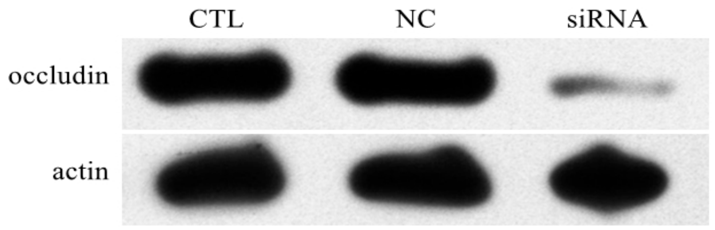

(a)

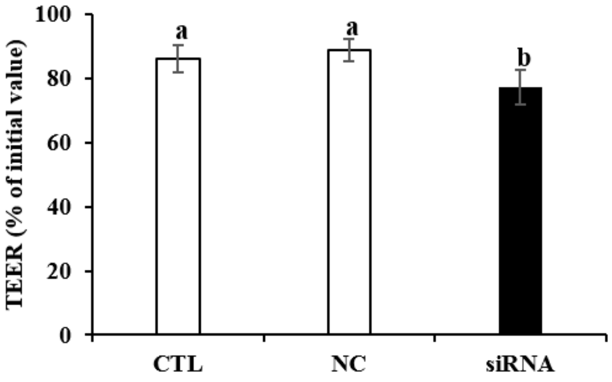

(b)

Figure S1. Mycotoxins disrupt intestinal epithelial permeability by affecting occludin expression. (**a**) Immunoblot of occludin in differentiated Caco-2 cells after transfection with occludin siRNA to validate the knockdown level. (**b**) Changes in TEER values in differentiated Caco-2 cells after occludin knockdown (siRNA) for 48 h compared with the initial value. Results are expressed as the mean ± S.E.M. of three independent experiments with three replicates. Different letters (a, b) indicate significant differences in TEER values ($p < 0.05$). NC, negative control.

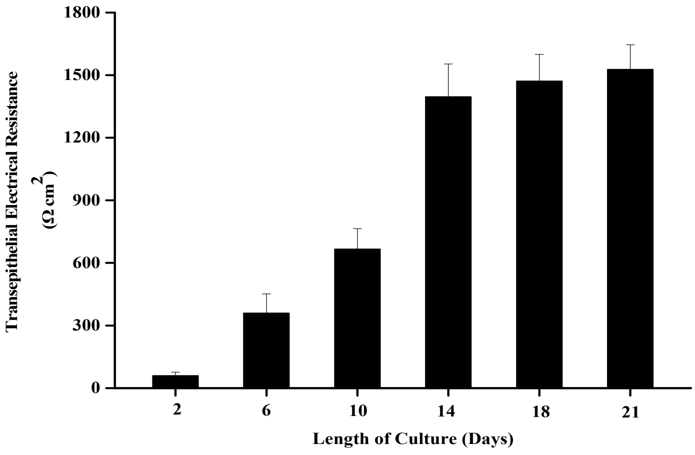

Figure S2. Transepithelial electrical resistance values (Ω × cm^2) in differentiated Caco-2 cells were measured at different time points until 21 days.

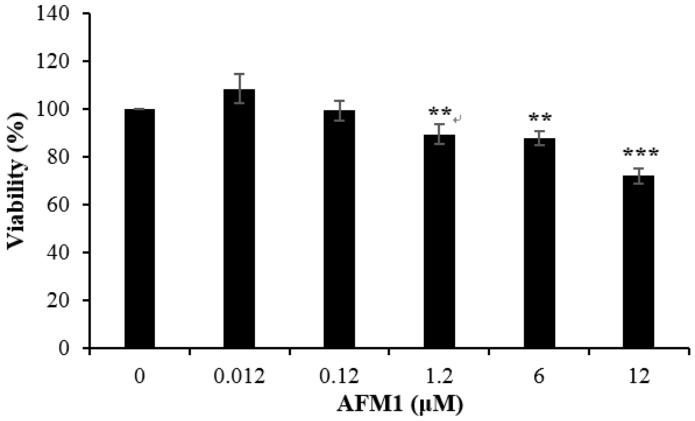

(a)

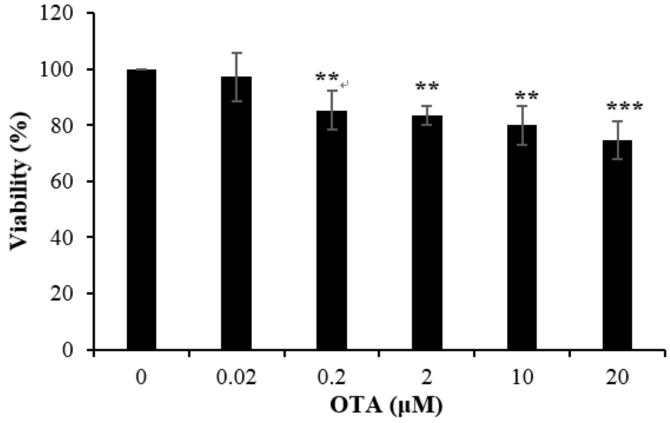

(b)

Figure S3. Concentration-response bar charts for AFM1 (**a**) and OTA (**b**) in Caco-2 cells after 48 h of exposure. Results are expressed as the mean ± S.E.M. of three independent experiments with five replicates. * $p < 0.05$; ** $p < 0.001$; and *** $p < 0.000$ indicates a significant difference from control cells.
